# Tribological Behavior of Hydraulic Cylinder Coaxial Sealing Systems Made from PTFE and PTFE Compounds

**DOI:** 10.3390/polym12010155

**Published:** 2020-01-07

**Authors:** Andrea Deaconescu, Tudor Deaconescu

**Affiliations:** Department of Industrial Engineering and Management, Transilvania University of Brasov, 500036 Brasov, Romania; deacon@unitbv.ro

**Keywords:** coaxial sealing systems, hydrodynamic friction, pressure distribution, virgin PTFE, filled PTFE

## Abstract

Current trends concerning hydraulic cylinder sealing systems are aimed at decreasing energy consumption which can be materialized by minimizing leaks and reducing friction. The latest developments in the field of materials and sealing system geometries as well as modern simulation possibilities allow maximum performance levels of hydraulic cylinders. Reducing friction is possible by hydro-dynamic separation of the sliding and sealing points already at very low velocities and by using materials, such as plastomers, from polytetrafluoroethylene (PTFE) (virgin PTFE and filled PTFE). It is within this context that this paper discusses a theoretical and experimental study focused on the tribological behavior of coaxial sealing systems mounted on the pistons of hydraulic cylinders. It presents a methodology for the theoretical determination of the lubricant film thickness between the cylinder piston and the seal. The experimental installation used for measuring fluid film thickness is presented, and the results obtained under various working conditions are compared to the theoretical ones. For the analyzed working conditions related to pressure, speed, and temperature, the paper concludes with a set of criteria for the selection of the optimum seal material so as to maximize energy efficiency.

## 1. Introduction

An important characteristic of hydraulic motors, whether linear or rotary, is their high energy density. Hence, a small-sized motor is capable of generating high power outputs. This benefit is due to the deployment working fluids at increasingly higher pressures (hundreds of bar) which requires rethinking the constructive and functional aspects of such motors. Thus, an important role is assigned to the sealing system.

A sealing system is defined as the assembly of elements designed to create the complete separation of two different media. The main component of such a system is the seal itself, a deformable or non-deformable element placed in a specially conceived seat. As the seal is pushed onto the sealed surface by a pre-tensioning force applied at mounting and/or by the fluid pressure, it achieves its function of rendering a hermetic system [1].

Conceiving an adequate sealing system requires information about seal geometry, the utilized materials, material–fluid compatibility, the quality of the contacting surfaces, sealing dynamics, etc. All these contribute to the energy efficiency of a sealing system, considering that high friction forces, fluid leaks or heat generation are responsible for the loss of energy that affects hydraulic systems. Studies conducted by ORNL/NFPA (Oak Ridge National Laboratory/National Fluid Power Association-USA) [2] have revealed an efficiency of merely 21% for hydraulic systems thus yielding the idea that, to date, this field has not been sufficiently optimized.

The energy efficiency of a linear hydraulic motor is increased by diminishing the friction forces in the sealing systems. Small friction forces can be ensured, however, only within certain limits, as reducing the contact pressure between the seal and the sealed surface automatically causes fluid leakage. The relationship between friction and pressure contact is influenced by the geometry and the material of the seal which entails the necessity of identifying a compromise solution between obtaining a reduced friction force and reduced leakage.

Most seals used in hydraulic drives are made from polymers, in particular from elastomers, plastomers or thermoplastic elastomers. The type of material that is utilized depends on the specific working conditions and its compatibility with the sealed fluid, fluid pressure, fluid temperature, etc. In order to ensure a long service life for sealing systems, the properties of seal materials need to be known in detail. The fact that, in many cases, contradicting characteristics are required, makes it difficult to identify and select the most adequate material.

Research concerning the construction and functioning of sealing system dates back over 80 years ago [3]. Reference [4] presents a historical review of the knowledge about the factors that affect the performance of hydraulic seals made from polymers.

The last years of the 20th century produced studies concerning the operational behavior of sealing systems made from elastomers. Thus, in 1987, Nau [5] presented a state-of-the-art study in the field of rubber seals. Bisztray-Balku [6] focused on the study of elastomers, where the author presented a critical analysis of the future of hydraulic systems and of the various developed tribological models. In addition, Nikas [7,8] analyzed the seals of rectangular cross-sections made from elastomers using a wide range of temperatures (−55 °C to 135 °C) and pressures (1 to 50 MPa).

As to the use of plastomers, materials which combine the qualities of elastomers and plastics, such as rubber-like properties with the processing ability of plastic [9], studies concerning their behavior can be found in numerous papers, the more relevant ones being References [10,11,12,13,14]. These papers reveal and discuss the phenomena of abrasive wear, the adherence of polytetrafluoroethylene (PTFE) seals, and the fatigue due to the high temperatures of the working fluid. Pure PTFE has limited applications as an engineering material due to the fact of its low wear resistance and cold flow. These limitations can be minimized by adding suitable fillers to a PTFE matrix to produce PTFE-based composites that are suitable for use as the friction units of technical equipment. The most common fillers used with PTFE are glass fiber, carbon fiber (CF), graphite, copper particle, molybdenum disulfide (Mo_2_S), and mixtures of these [15]. Fillers, such as bronze, glass fiber, Mo_2_S or carbon, added into the composition of PTFE influence seal behavior in different ways. Thus, for example, while bronze improves wear resistance at the temperature of the surrounding environment, its effect is opposite at high temperatures.

Over the last years, many studies have presented results on the regime of friction in the sealing tribological system. It was revealed that, in the case of certain dynamic sealing systems, the occurrence of a relative motion between the seal and its contact surface causes a thin fluid film to be built up between the two elements. Müller [16] addressed the dependency of lubricant film thickness on its viscosity as well as the compressibility of seals in mounting. A conclusion of Müller’s research stated that lubricant film thickness is not the same for the two directions of motion of the friction couple mobile element.

The results of other research concerning the character of friction in the sealing tribological system are described in the works of Kanzaki et al. [17], Stupkiewicz et al. [18], Fatu et al. [19], Crudu et al. [20]. It needs pointing out, however, that those works refer only to concrete cases of sealing systems without the possibility of generalization.

The present paper analyzed the mechanism and character of friction in coaxial sealing systems made from PTFE and its compounds. The use of these materials was justified by their low friction properties that ensure a high level of energy efficiency. The study’s focus on sealing systems was determined by their widespread use, constructive simplicity, and the polymers they are made from—materials with pronounced low friction properties.

The paper is structured as follows: Section 2 of the paper describes the structure of a coaxial sealing system and presents the distribution of pressures in the seal–sealed surface contact area. The onset of piston motion favors the occurrence of a gap between the seal and the cylinder wall. It is the magnitude of such gap that determines the type of friction within the sealing system. The paper further specifies the hypotheses that underlie the equation for computing the gap. Section 3 and Section 4 of the paper present the theoretical and experimental results of testing three different polymeric materials. The results reveal the dependency of the type of friction on the pressure and temperature of the working fluid as well as on the relative velocity among the elements of the sealing system. The last section of the paper is comprised of the conclusions yielded by the study.

## 2. The Mechanism of Sealing

**Figure 1:**
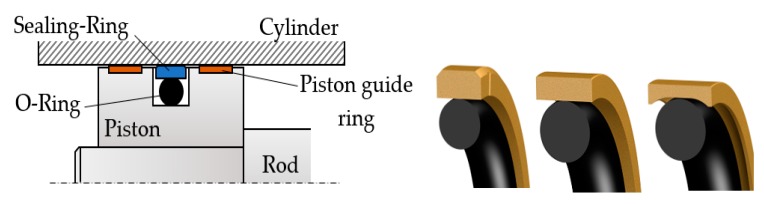
Coaxial sealing system of a piston.

Coaxial sealing systems are assemblies consisting of a seal made from a material with advanced low friction properties and an O-ring that ensures the pre-tensioning of the entire package. Generally, the seal is made of polytetrafluoroethylene (virgin or filled PTFE) and the O-ring of elastomers of various types: nitrile butadiene rubber (NBR), fluorocarbon (FKM), ethylene propylene diene monomer (EPDM), hydrogenated nitrile butadiene rubber (HNBR), fluorosilicone (FVMQ), and silicone rubber (Q). Figure 1 shows an example of a coaxial sealing system used for a piston and several cross-section forms of the seal [21].

In order to achieve the sealing effect, a radial pressure has to be exerted upon the seal (sealing ring) by means of the O-ring. The O-ring is placed into its seat in a pre-tensioned state with an initial specific radial deformation *ε_r_*_0_ of 10% to 25%. Upon the onset of fluid pressure inside the hydraulic motor, the O-ring is deformed additionally and exerts a greater radial pressure.

A relative motion occurring between the seal and the inner surface of the cylinder generates, according to the laws of hydrodynamic lubrication, a dynamic pressure and a fluid film of variable thickness. Depending on the velocity (*v*) of the piston, the dynamic viscosity (*η*) of the working fluid, and the compressibility of the seal material, the friction between the seal and the cylinder surface can be of dry, fluid or mixed type. Fluid friction ensures a good energy efficiency due to the diminished friction forces, a situation nevertheless conflicting with the phenomenon of sealing. The hydrodynamic separation between the seal and the surface of the cylinder that is typical for fluid friction causes increased fluid loss by leakage, as the fluid flows towards the lower pressure side.

While the presence of a gap (*g*) has the beneficial effect of diminishing the friction forces, its inconvenience is leakage. The effect of inadequate sealing is a certain fluid loss that can manifest as *leakage* (the Poiseuille component of flow) or *drag* (the Couette component of flow). Leakage means fluid loss, even at rest, caused by the pressure drop between the two sealed chambers. Fluid drag is determined by the existence on the moving component of a fluid film that is necessary for ensuring minimum friction forces [22]. Evidently, it is the fluid volume lost by drag that is of interest in the study of sealing systems, leakage being specific only to defect seals.

**Figure 2:**
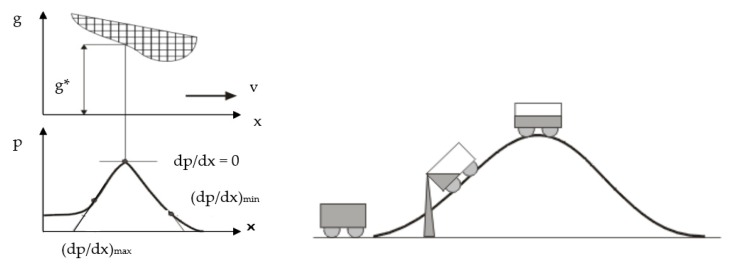
Evolution of a pressure in the gap and the Prokop analogy.

The thickness of the fluid film between the seal and the cylinder surface is determined by the evolution of the pressure gradient in the gap (*dp*/*dx*): a large gradient means a thin fluid film in the sealed area, while a small gradient determines a thicker fluid film. Figure 2 illustrates the evolution of the fluid film in the contact area of the seal and cylinder surface, known as the Prokop analogy [23]. It can be noticed that the gradient of the curve determines the volume of the leaked fluid. A greater gradient (*dp*/*dx*) causes a smaller quantity of dragged fluid.

For a viscous flow, the basic relationship between the pressure gradient (*dp*/*dx*) and the dimension of the gap, *g*(*x*), in the direction of motion *x* while neglecting inertial forces is [24]:

(1) $$\frac{dp}{dx}=6\cdot \eta \cdot v\cdot \frac{g\left(x\right)-\;g^{*}}{g^{3}\left(x\right)}$$

where g* is the dimension of the gap at the point of zero pressure gradient (*dp*/*dx* = 0).

Crucial for assessing the type of friction in a coaxial sealing system is determining the magnitude and evolution of the gap (*g*) formed between the seal and its contact surface.

**Figure 3:**
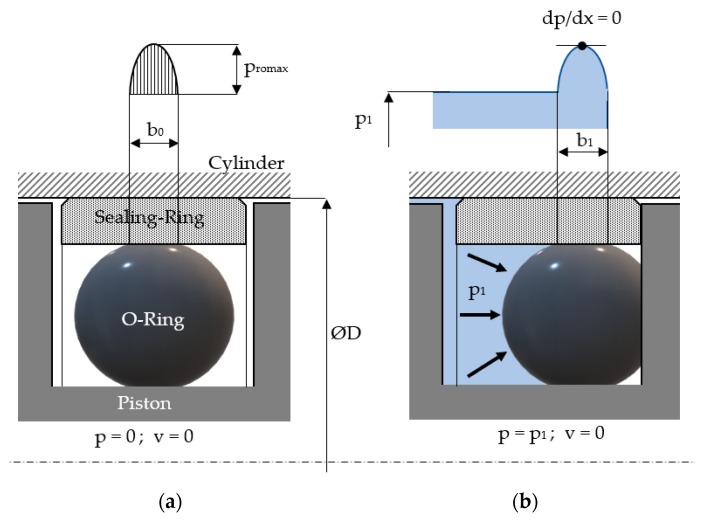
Distribution of pressure at the contact between the seal and the cylinder surface (*v* = 0) (**a**) in the absence and (**b**) presence of fluid pressure.

Figure 3 shows at rest the distribution of the contact pressure at the interface of the seal and the cylinder surface in two cases: (a) in the absence and (b) presence of fluid pressure.

Upon being mounted in its seat, the O-ring modifies its geometry. It is deformed in two directions: radially and axially. Of interest for the study of the mechanism of friction is only the radial deformation that influences the magnitude of the gap between the seal and the cylinder surface.

At rest and in the absence of the sealed pressure (Figure 3a), the distribution of the radial pressure *p_r_*_0_(*x*) on the sealed surface is a parabola described by Equation (2) [24]:

(2) $$p_{r0}=p_{r0max}\cdot \sqrt{1-{\left(2\cdot \frac{x}{b_{0}}-1\right)}^{2}}$$

where *b*_0_ is the width of the contact surface between the O-ring and the seal, and *p_r_*_0*max*_ is the maximum contact pressure given by Equation (3) [24]:

(3) $$p_{r0max}=\frac{\sqrt{\varepsilon_{r0}^{2}\cdot \left(m_{r}^{2}-1\right)+2\cdot \varepsilon_{r0}\cdot \left(m_{r}+1\right)}\cdot \left(\frac{H}{4}+\frac{H^{4}}{10^{6}}\right)}{2\cdot \left(1-m_{r}^{2}\right)}$$

All notations used in Equation (3) refer to the O-ring: *m_r_* is Poisson’s ratio for the ring material (NBR), *ε_r_*_0_—is its initial specific radial deformation, and *H* is the Shore A hardness of the material.

In Figure 3b the cylinder is fed a pressure *p*_1_, in which case, in the absence of a relative velocity *v*, the distribution of pressure on the sealed surface is given by Equation (4) [24]:

(4) $$p\left(x\right)=p_{1}+3\cdot \frac{\eta \cdot v\cdot L\cdot \left(1-\beta \right)}{g_{0}^{2}}\cdot \left[1-\frac{L\cdot \left(1-\beta \right)}{2\cdot x}\right]$$

with the following notations: *β = Ar*/*An* = the real non-dimensional contact area; *An, Ar* = the nominal, real contact area, respectively; *v* = gliding velocity; *L* = width of the seal; *g*_0_ = fluid film thickness at zero pressure gradient.

The real non-dimensional contact area is less than unity and is a quantity that accounts for the materials of the two elements of the friction pair (i.e., seal and cylinder), the initial specific radial deformation *ε_r_*_0_ of the O-ring, and the pressure of the working fluid.

**Figure 4:**
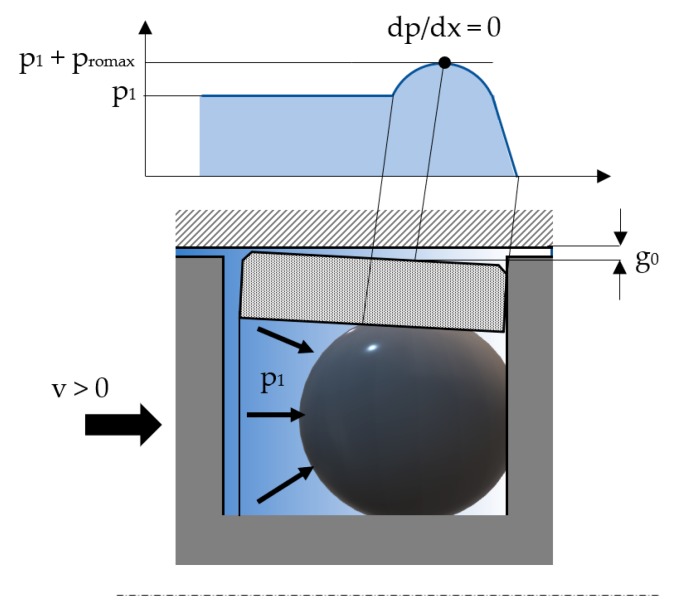
Distribution of pressure at the contact between seal and cylinder surface (v ≠ 0).

While at rest, the seal and the cylinder are in direct contact; upon onset of motion, the two elements will be completely separated by a “wedge”-shaped gap (Figure 4).

It is the radial elastic deformation of the seal that allows the generation between the two surfaces of a thin fluid film. The separation of the two surfaces is caused by the dynamic component of the developed pressure given by Equation (5) [24]:

(5) $$p_{d}\left(x\right)=p_{1}+3\cdot \frac{\eta \cdot v\cdot L\cdot \left(1-\beta \right)}{g_{0}^{2}}\cdot \left[1-\frac{L\cdot \left(1-\beta \right)}{2\cdot x}\right]$$

The fluid flow through the thus formed gap can be studied starting from several hypotheses:

- The deformation of the seal (the magnitude of the gap) is small compared to the pre-tensioning of the O-ring at mounting;
- The thickness of the fluid film in the gap is small compared to the radius of the seal;
- It is admitted that the pressure distribution in the gap is identical to that determined for the O-ring;
- It is admitted that, on radial direction, a balance appears between the pressure created by the compression of the O-ring and the dynamic component of the pressure in the gap. The two pressures are of equal magnitude and the form of the gap is determined by the distribution of pressure generated by the O-ring.

The calculation of the magnitude of the gap formed between the seal and the cylinder surface is based on the hypothesis that the seal is a cylinder with thin walls subjected to a pressure given by Equation (5). Thus, at zero pressure gradient, the gap *g*_0_ is [25,26]:

(6) $$g_{0}=\;\sqrt[{3}]{\frac{3}{16}\cdot \frac{{\left(D-h\right)}^{2}}{E_{p}\cdot h}\cdot \eta \cdot v\cdot L\cdot \left(1-\beta^{2}\right)\cdot \left[1-\frac{2\cdot \cosh\left(k\cdot L\right)\cdot \cos\left(k\cdot L\right)}{\cosh^{2}\left(k\cdot L\right)+\cos^{2}\left(k\cdot L\right)}\right]}$$

where:

(7) $$k=\;\sqrt[{4}]{\frac{12\cdot \left(1-m_{p}^{2}\right)}{h^{2}\cdot {\left(D-h\right)}^{2}}}$$

where *m_p_* and *E_p_* are Poisson’s ratio and Young’s modulus of the seal material, respectively; *h* = thickness of the seal.

Equation (6) highlights the direct dependency of the fluid film thickness on the velocity, the viscosity of the working fluid, and the seal width. Thus, the velocity influences decisively the type of friction in the coaxial sealing system. In the absence of motion among the components of the tribosystem (at the debut of motion), friction is dry due to the adhesion forces among the contacting materials. Upon the onset of the motion, the adhesion forces, the internal friction forces as well as the forces caused by the asperities of the two surfaces clinging one to another determine mixed friction. As the velocity grows, the velocity of the fluid determines the complete separation of the tribosystem components (known as grease planning) which indicates the presence of fluid (hydrodynamic) friction [25].

Generally, fluid friction appears only when the thickness of the fluid film is at least equal to the sum of the roughness values *R_max_* of the two surfaces initially contacting. In the case of coaxial sealing systems, the conducted experimental research has revealed that fluid friction is present even when the average thickness of the fluid film falls below the sum of the roughness values *R_max_* of the two surfaces [26]. This is favored by the fact that the seal material, being softer than the material of the hydraulic cylinder, adapts its form to the asperities of the cylinder’s steel surface [26].

The viscosity of the working fluid also influences the magnitude of *g*_0_ and, consequently, the type of friction. The decreasing of the viscosity, due to the work temperature increase, diminishes the thickness of the fluid film which can affect the type of friction.

## 3. Theoretical results

In order to determine the type of friction in coaxial sealing systems, the test included seals made of three different polymeric materials of the category of polytetrafluoroethylenes (virgin PTFE and filled PTFE). In all cases, the O-ring was made of nitrile butadiene rubber (NBR) of 70 Shore A hardness. Table 1 features the characteristics of the studied materials [27].

Polytetrafluoroethylene has one of the smallest friction coefficients ever recorded in a solid material (0.05 to 0.1). As it includes high-bonded carbon and fluorine, PTFE is almost completely inert to the substances it comes into contact with. These two properties account for the successful use of this material for tribological applications designed for reducing energy consumption in friction-intensive machinery as well as for reactive and corrosive applications.

The added carbon fibers increase wear resistance, reduce the friction coefficient, and improve the thermal expansion properties. The addition of bronze to PTFE improves compression strength, thermal conductivity, and electrical conductivity. Also reduced is the tendency to extrusion while maintaining good sliding and wear properties. The PTFE-ul with added bronze is the standard material in hydraulic applications [28].

**Figure 5:**
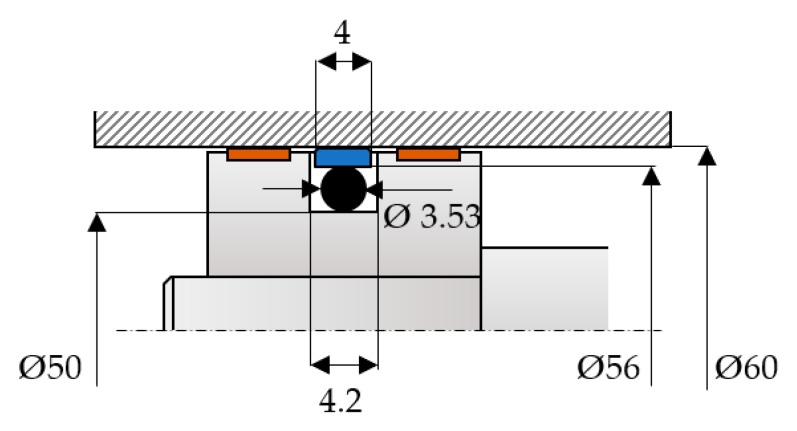
Dimensions of the tested coaxial sealing system.

Figure 5 presents the dimensions of the studied coaxial sealing systems.

**Figure 6:**
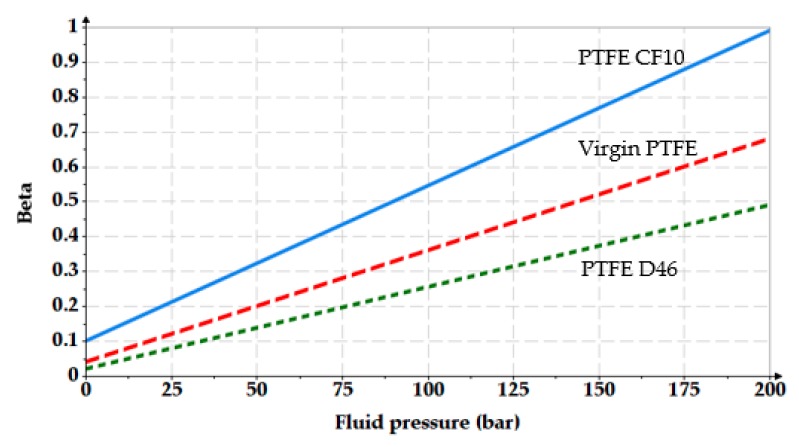
Dependency of the real non-dimensional area on the seal material and on the sealed pressure.

An initial specific radial deformation *ε_r_*_0_ of 15% resulted for the dimensions of the O-ring seat. The real non-dimensional area *β* for the friction pair consisting of the steel cylinder and the three seals made of different materials depends on the initial specific radial deformation *ε_r_*_0_ of the O-ring and on the fluid pressure. Figure 6 shows these dependencies.

The fluid used for testing was anti-wear hydraulic oil ISO VG 32 which is a premium light-weight paraffinic-based hydraulic oil, ideal for industrial applications or for hydraulic systems [29]. Equation (8) describes the influence of temperature on the dynamic viscosity of the working fluid:

(8) $$\eta =A\cdot e^{\frac{B}{T}}$$

where *A* = 5.68 × 10^−9^ and *B* = 4827.627 [29].

**Figure 7:**
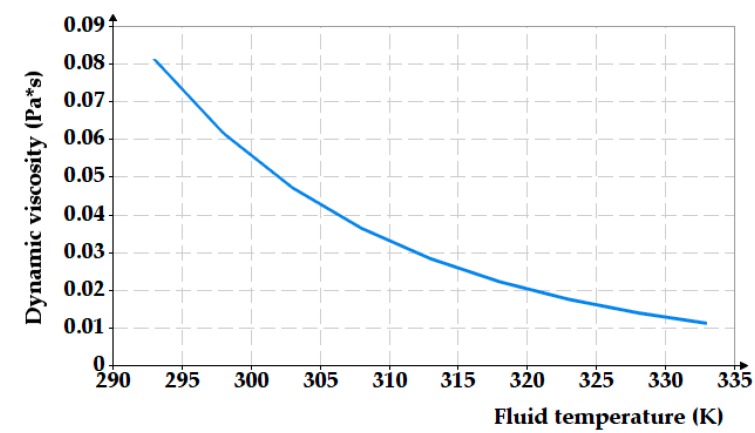
Dependency of the dynamic viscosity on the temperature of the sealed fluid.

For oil temperatures between 20 °C and 60 °C (293 to 333 K), Figure 7 shows the variation of the dynamic viscosity versus temperature.

**Figure 8:**
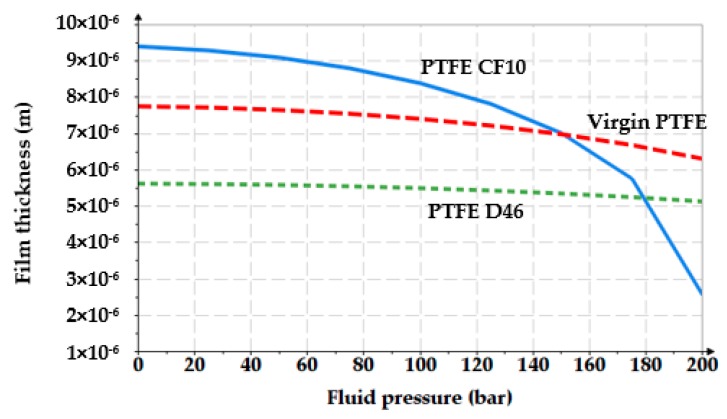
Variation of the fluid film thickness versus the working pressure.

The influence of the working fluid pressure on the thickness of the fluid film was analyzed for an oil temperature of 60 °C (333 K) which implies a dynamic viscosity of 0.011 Pa∙s. The considered velocity was of 0.2 m/s. Figure 8 shows the resulting graph.

**Figure 9:**
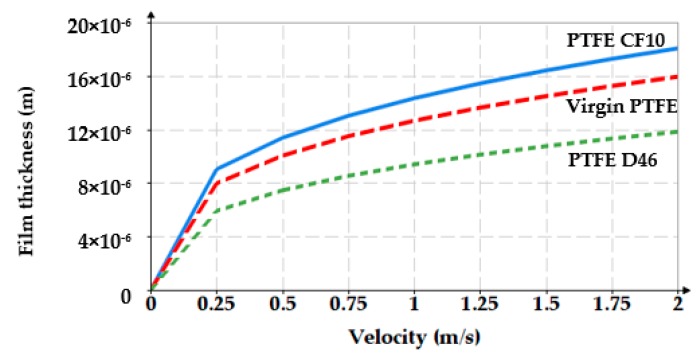
Variation of the fluid film thickness versus piston velocity.

For an oil pressure of 100 bar at a temperature of 60 °C (333K), Figure 9 shows the *g*_0_ *= f*(*v*) diagram.

**Figure 10:**
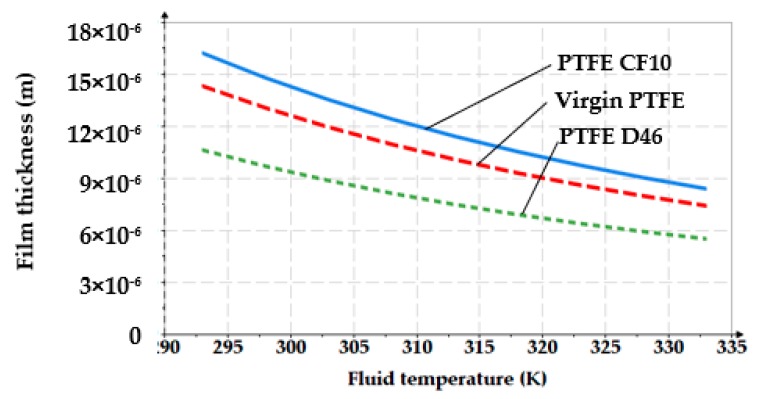
Variation of the fluid film thickness versus fluid temperature.

The variation of the working fluid temperature also causes modifications of the fluid film. For a pressure of 100 bar and a velocity of 0.2 m/s, Figure 10 shows the *g*_0_ *= f*(*T*) graph.

**Figure 11:**
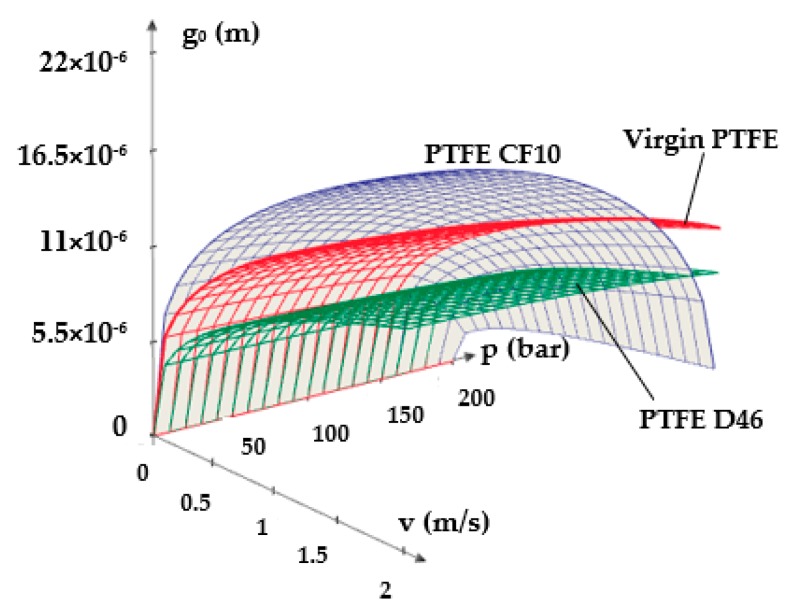
Variation of the fluid film thickness versus working velocity and pressure.

**Figure 12:**
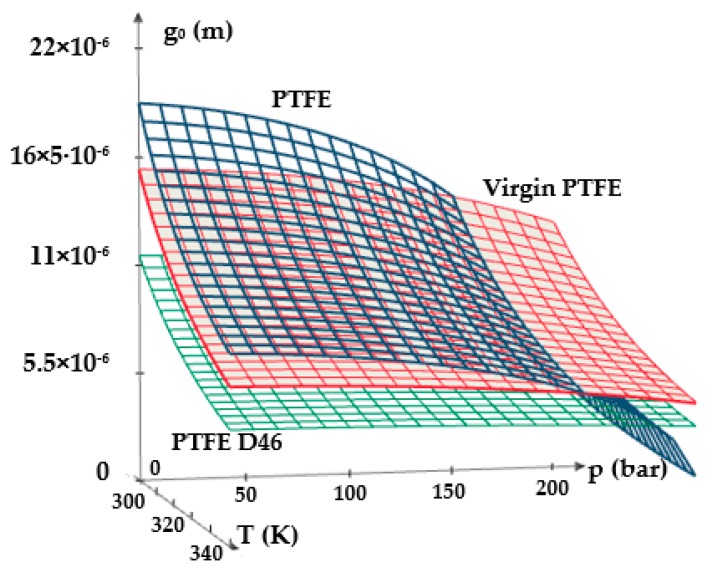
Variation of the fluid film thickness versus pressure and temperature.

Figure 11 and Figure 12 show 3D representations of the fluid film thickness variation versus pressure, working velocity, and temperature.

**Figure 13:**
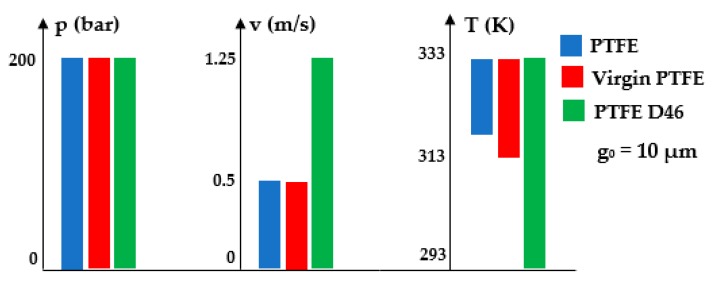
Utilization limits of the various materials.

The above figures yield a series of conclusions:

- While with increasing pressure a decrease of *g*_0_ can be noticed, for the analyzed interval from 0 to 200 bar, the diminishing of the fluid film was, however, rather small (of approximately 1 μm). For PTFE with carbon fibers (PTFE CF10) at high working pressures, the thickness of the fluid film decreased significantly up to 6.5 μm which worsens the conditions of friction;
- The thickness of the fluid film grows with increasing velocities. The presence of a consistent fluid film at the seal–cylinder interface causes a significant decrease of friction forces and seal wear. On the other hand, high velocities carry the risk of fluid drag and, consequently, of inadequate sealing;
- The increase of the sealed fluid temperature causes the diminishing of its dynamic viscosity which determines a diminishing of the fluid film thickness. This triggers an unfavorable friction type;
- The fluid film thickness has micrometric values (1 to 20 μm). As the recommended maximum roughness (the maximum peak-to-valley height) of hydraulic cylinder interior surfaces is of *R_max_* = 0.63 to 2.5 μm [30], under certain working conditions, the thickness of the fluid film *g*_0_ is greater than the roughness sum of the surfaces that form the friction pair which yields the conclusion that fluid (hydrodynamic) friction is dominant. If the maximum admissible limit of the fluid film thickness is set to *g*_0_ = 10 μm, where fluid drag is within acceptable limits for pistons, Figure 13 presents recommendations for the selection of the seal material. A maximum thickness of 10 μm is imposed also because, correspondingly, the fluid still has a laminar flow in the gap, while at higher thickness values, the flow turns turbulent which causes undesirable friction losses [31].

Figure 13 shows that any of the three materials can be used in the imposed pressure range. As regarding the velocity and temperature conditions, the most adequate material to be used is PTFE 46D with added bronze.

## 4. Experimental Results

**Figure 14:**
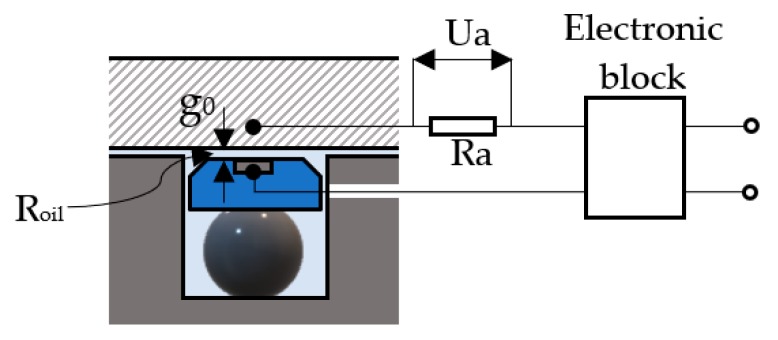
Measuring configuration.

As the fluid film thickness cannot be measured directly, this was achieved indirectly by the resistive method. This requires measuring the voltage drop on the resistance created by the fluid film between the seal and the surface of the hydraulic cylinder. The electrical connections to the sealing system are shown in Figure 14 [23].

Resistance *R_oil_* is determined by fluid film of thickness *g*_0_, and *Ra* is the resistance of the utilized measuring device (*Ra* = 10 MΩ). In the presented measuring configuration, the electrical resistance *R_oil_* is computed by Equation (9):

(9) $$R_{oil}=10\cdot \frac{10V\;-\;Ua}{Ua}\;\;\;\left(M\Omega \right)$$

The dependency between resistance *R_oil_* of the fluid film and its thickness *g*_0_ is linear [22]:

(10) $$C_{R}=\;\frac{R_{oil}}{g_{0}}=63.735\left(M\Omega /µm\right)$$

from where follows the computational relationship of the fluid film thickness:

(11) $$g_{0}=\;\frac{Ra\cdot \left(10V-Ua\right)}{C_{R}\cdot Ua}\cdot 10^{-6}\;\;\left(m\right)$$

Table 2 presents the experimental results obtained with this setup.

**Figure 15:**
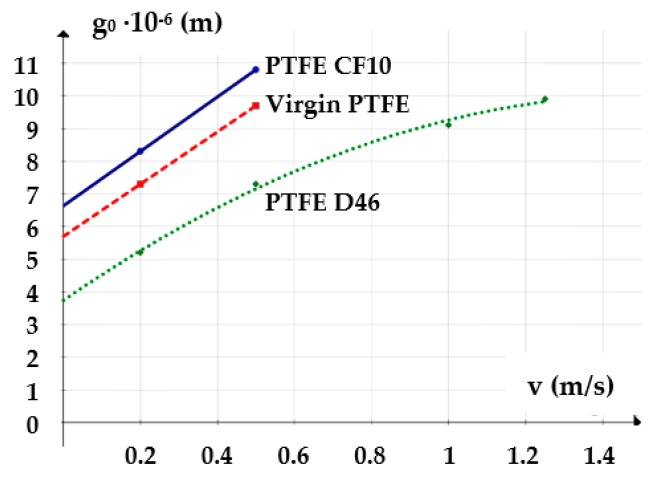
Variation of the fluid film thickness versus working velocity (experimental).

**Figure 16:**
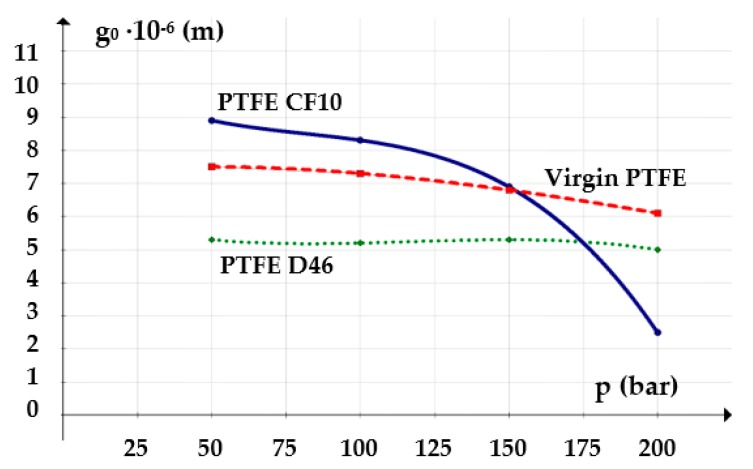
Variation of the fluid film thickness versus working pressure (experimental).

**Figure 17:**
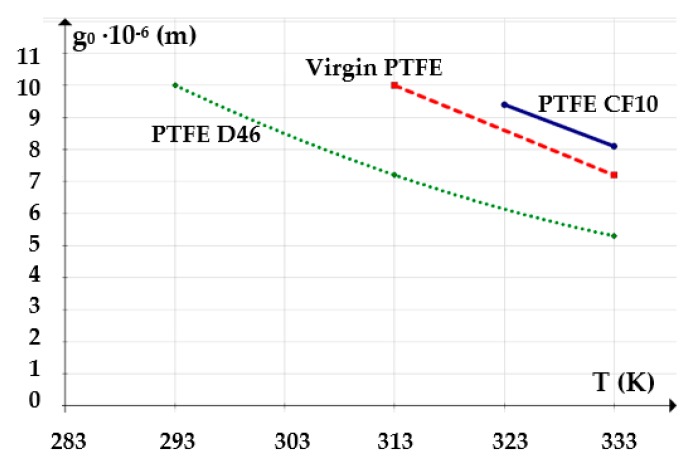
Variation of the fluid film thickness versus fluid temperature (experimental).

The graphs in the following figures (Figure 15, Figure 16 and Figure 17) present the dependencies of the fluid film thickness on the velocity, fluid pressure, and temperature resulting from the conducted experiments.

The analysis of the three graphs shows that the measured values were very close to those calculated by Equation (6) with a maximum error of 3.5%. Thus, the conclusions of the theoretical research were confirmed, namely, that the thickness of the fluid film grows with the increasing velocity and is diminished as the pressure and temperature of the sealed fluid increase.

## 5. Conclusions

The paper analyzes the operational behavior of three seals made of PTFE-based materials, one of 100% PTFE concentration (virgin PTFE), and two others with 10% carbon fibers and 46% bronze, respectively.

The sealing mechanism was explained with a focus on the fact that, at the onset of a certain velocity, the elements of the friction pair are separated by a fluid film of a certain thickness. The presence of the fluid film causes hydrodynamic friction.

The theoretical and experimental studies yielded a series of significant conclusions:

- The thickness of this film grows with increasing relative velocity;
- With increasing working pressure, between the seal and its adjacent surface, dry contact areas exceed the hydro-dynamically separated ones;
- Higher working fluid temperature and pressure cause smaller film thicknesses;
- Of the three tested materials, the most adequate for utilization is PTFE 46D with added bronze;
- Virgin PTFE and PTFE CF10 (with added carbon fibers) are adequate for small velocities and relatively high temperatures;
- At high working pressures, seals made of PTFE CF10 deform less, and a sudden decrease of the fluid film occurs with adverse effects on friction;
- The theoretically determined computational relationship for the fluid film thickness has been confirmed by the experimental results.

The research results yielded the conclusion that the utilization of coaxial sealing systems with seals made of PTFE 46D is the recommended solution for the construction of linear hydraulic motors.

## Figures and Tables

**Table 1:** Characteristics of the seal materials.

Material	Composition	Sh D Hardness	Young’s modulus (MPa)
PTFE CF10	90% PTFE + 10% carbon fiber	58 ± 3	300
Virgin PTFE	100% PTFE	55 ± 3	540
PTFE D46	53% PTFE + 46% bronze + 1% pigments	63 ± 3	1420

**Table 2:** Measured voltage drops and corresponding computed fluid film thicknesses.

Material	*Ua* (V)	*g*_0_ (μm)	*Ua* (V)	*g*_0_ (μm)	*Ua* (V)	*g*_0_ (μm)	*Ua* (V)	*g*_0_ (μm)
	*p* = 100 bar; *T* = 333 K							
	*v* = 0.2 m/s		*v* = 0.5 m/s		*v* = 1 m/s		*v* = 1.25 m/s	
CF10	0.186	8.3	0.143	10.8	-	-	-	-
PTFE	0.21	7.3	0.159	9.7	-	-	-	-
D46	0.293	5.2	0.21	7.3	0.169	9.1	0.156	9.9
	***v* = 0.2 m/s; *T* = 333 K**							
	***p* = 50 bar**		***p* = 100 bar**		***p* = 150 bar**		***p* = 200 bar**	
CF10	0.173	8.9	0.186	8.3	0.222	6.9	0.591	2.5
PTFE	0.205	7.5	0.21	7.3	0.226	6.8	0.251	6.1
D46	0.288	5.3	0.293	5.2	0.288	5.3	0.304	5
	***p* = 100 bar; *v* = 0.2 m/s**							
	***T* = 293 K**		***T* = 313 K**		***T* = 333 K**			
CF10	-	-	-	-	0.19	8.1		
PTFE	-	-	0.154	10	0.21	7.2		
D46	0.154	10	0.213	7.2	0.288	5.3		
